# Pathways of copper import and utilization that support respiration in *Bacillus subtilis*

**DOI:** 10.1128/mbio.01069-26

**Published:** 2026-06-09

**Authors:** Grayson Barnes, Lars Hederstedt, Oriana S. Fisher, Claes von Wachenfeldt, John D. Helmann

**Affiliations:** 1 Department of Microbiology, Cornell University 251789, Ithaca, New York, USA; 2 The Microbiology Group, Department of Biology, Lund University 193193, Lund, Sweden; 3 Department of Molecular Biology and Biochemistry, Wesleyan University 5468, Middletown, Connecticut, USA; NYU Langone Health, New York, New York, USA

**Keywords:** *Bacillus subtilis*, copper, respiration, transporters, multicopper oxidases, metal resistance

## Abstract

Copper is an enzyme cofactor that is essential for cellular respiration in most aerobic organisms but can be toxic in excess. Although most copper enzymes are localized within or external to the cell membrane, proper copper loading often relies on copper import into the cytosol. In *Bacillus subtilis*, the YcnJ membrane protein has been proposed to serve as a copper importer, but its biological function remains poorly characterized. Here we demonstrate that under copper starvation, YcnJ is important for aerobic growth by supporting the proper function of Qox, the major respiratory menaquinol oxidase (cytochrome *aa*_3_). YcnJ function requires a Cu^2+^-binding site in an extracellular CopC domain and a second putative copper-binding site in its transmembrane CopD domain. YcnJ additionally contains a carboxy-terminal YtkA domain with homology to the membrane-anchored lipoprotein CtaK that has been implicated in the assembly of the Cu_A_ center in the *caa_3_*-type cytochrome oxidase Cta. The function of Cta is compromised in a *ctaK* mutant, and this effect is exacerbated if YcnJ or its C-terminal YtkA domain is absent. We further show that during copper starvation, inactive heme-containing Qox and CtaA accumulate in the membrane and that under copper excess, CtaM is involved in copper resistance and oxidase assembly. These findings refine current models of copper import and trafficking in *B. subtilis* as it supports the function of respiratory heme-copper oxidases.

**IMPORTANCE:**

Copper is a critical micronutrient for bacteria; however, it is toxic in excess. This leads to the cell having tightly regulated import and export systems to prevent intoxication while having sufficient copper for cellular needs. Copper export has been well studied, but copper import and the effects of copper starvation are less defined. In this article, we characterize the *Bacillus subtilis* high-affinity copper importer YcnJ. We show that under copper limitation, the cell is deficient in copper-containing oxidases, which leads to respiratory defects. Copper imported by YcnJ is proposed to function in a pathway with other proteins implicated in the assembly of respiratory oxidases. More generally, this work contributes to the underexplored area of copper import and trafficking in bacteria.

## INTRODUCTION

Copper (Cu) is an essential micronutrient for most living organisms. Bioinformatic surveys reveal that known copper-dependent enzymes are encoded in 80% of bacterial, 50% of archaeal, and >95% of eukaryotic genomes (1). Although less than 1% of enzymatic reactions require copper, some of these processes are essential to cellular physiology (2, 3). Copper is especially important in aerobic respiration, where it serves as a redox center in cytochrome *c* oxidases and some quinol oxidases (4, 5).

Copper has the strongest affinity for ligands among intracellular metal ions, as reflected by its position at the top of the Irving-Williams series (6). As a result, there are no free (hydrated) Cu^+^ ions in the cell (7), and metalation of copper-requiring proteins is often facilitated by direct transfer from dedicated protein chaperones (8–10). Most copper proteins are in the cell membrane, periplasm, or extracellular space. For many copper enzymes, copper import is required for efficient protein metalation (11). Metalation may then occur in the cytosol, either during or after protein synthesis, or after translocation of the apo-protein from the cytosol to the membrane or extracellular space. In the latter case, metalation may involve both copper import and copper export proteins, although direct metalation from the environment may sometimes occur (11, 12).

Copper is ubiquitously found as an enzyme cofactor in terminal oxidases within the respiratory chain (4, 5). The *Bacillus subtilis* genome encodes three terminal oxidases, two of which require Cu for their activity (13). The major respiratory oxidase is the heme-copper enzyme cytochrome *aa*_3_ (Qox), which is a menaquinol oxidase made up of four protein subunits and contains a mononuclear Cu_B_ center. Mutants lacking Qox exhibit a growth defect on non-fermentable substrates under aerobic conditions. The second terminal oxidase is the cytochrome *c*-dependent heme-copper enzyme cytochrome *caa*_3_ (Cta). Cta contains four protein subunits with a binuclear Cu_A_ center in CtaC and a mononuclear Cu_B_ site in CtaD (13). Assembly of Cta is known to involve numerous accessory factors, including CtaG, CtaM, CtaK, and Sco (also named YmpQ) (4, 14, 15). Mutant cells defective in Cta grow well and are most conveniently identified by staining for N,N,N′,N′-tetramethyl-p-phenylenediamine dihydrochloride (TMPD)-oxidase activity (16). The third terminal oxidase, cytochrome *bd* (Cyd), is a copper-independent menaquinol oxidase. Expression of Cyd is repressed by the transcription factor Rex and induced under microaerobic conditions (17). Cyd activity can be assessed genetically since it is essential for aerobic growth in the absence of Qox (18). In addition to the Cta and Qox oxidases, the only other known cuproenzyme in *Bacillus* is a multicopper laccase (CotA) assembled into the spore coat during sporulation (19).

Intracellular copper levels are tightly regulated by import and export systems that are regulated by metal-sensing transcription factors (20, 21). Despite being indispensable for many organisms, copper can also be extremely toxic (22, 23). The high affinity of cupric ion (Cu^+^), thought to be the dominant form in the intracellular milieu, to biomolecules can lead to non-specific binding to proteins and enzymes, rendering them non-functional (24, 25). In *B. subtilis,* Cu^+^ export is mediated by the P-type ATPase CopA and assisted by the Cu^+^ chaperone CopZ (26–28). The *copZA* operon is repressed by the Cu^+^-sensing CsoR metalloregulator (29). Like other metal-transporting P-type ATPases, the *B. subtilis* CopA contains multiple N-terminal metal-binding domains, in addition to its catalytic domain (27, 30, 31). CopA family exporters often function with other partner proteins to protect cells against copper toxicity (22, 32).

In contrast to copper export, bacterial copper import pathways are poorly characterized. The requirement for copper to support bacterial growth is low, with ~10^3^ to 10^4^ atoms per cell when grown in minimal medium (MM) (33), and bacteria often have high-affinity uptake systems. As a result, physiological studies typically rely on copper chelators to impose copper limitation. In *B. subtilis*, copper import has been proposed to require the *ycnKJI* operon (33, 34). YcnK encodes a transcriptional repressor that regulates the *ycnKJI* operon, which additionally encodes a postulated copper importer YcnJ and the DUF1775 family Cu^2+^ chaperone YcnI (33–35). Both YcnJ and YcnI have extracellular Cu^2+^-binding domains that allow intermolecular copper transfer (36), and YcnJ has a transmembrane CopD domain proposed to import Cu^2+^ (33).

In this study, we find that YcnJ can support the function of both Qox and Cta. We show that, under copper limitation, a ∆*ycnJ* strain exhibits a severe growth defect due to impaired Qox function. Epistasis experiments and bioinformatic analyses suggest that CtaM, known to be important for the functional assembly of both copper-containing oxidases in *B. subtilis* (15), may be involved in export of cytosolic copper to support assembly of both Qox and Cta.

## RESULTS

### YcnJ and YcnI support growth under copper-limiting conditions

**Fig 1:**
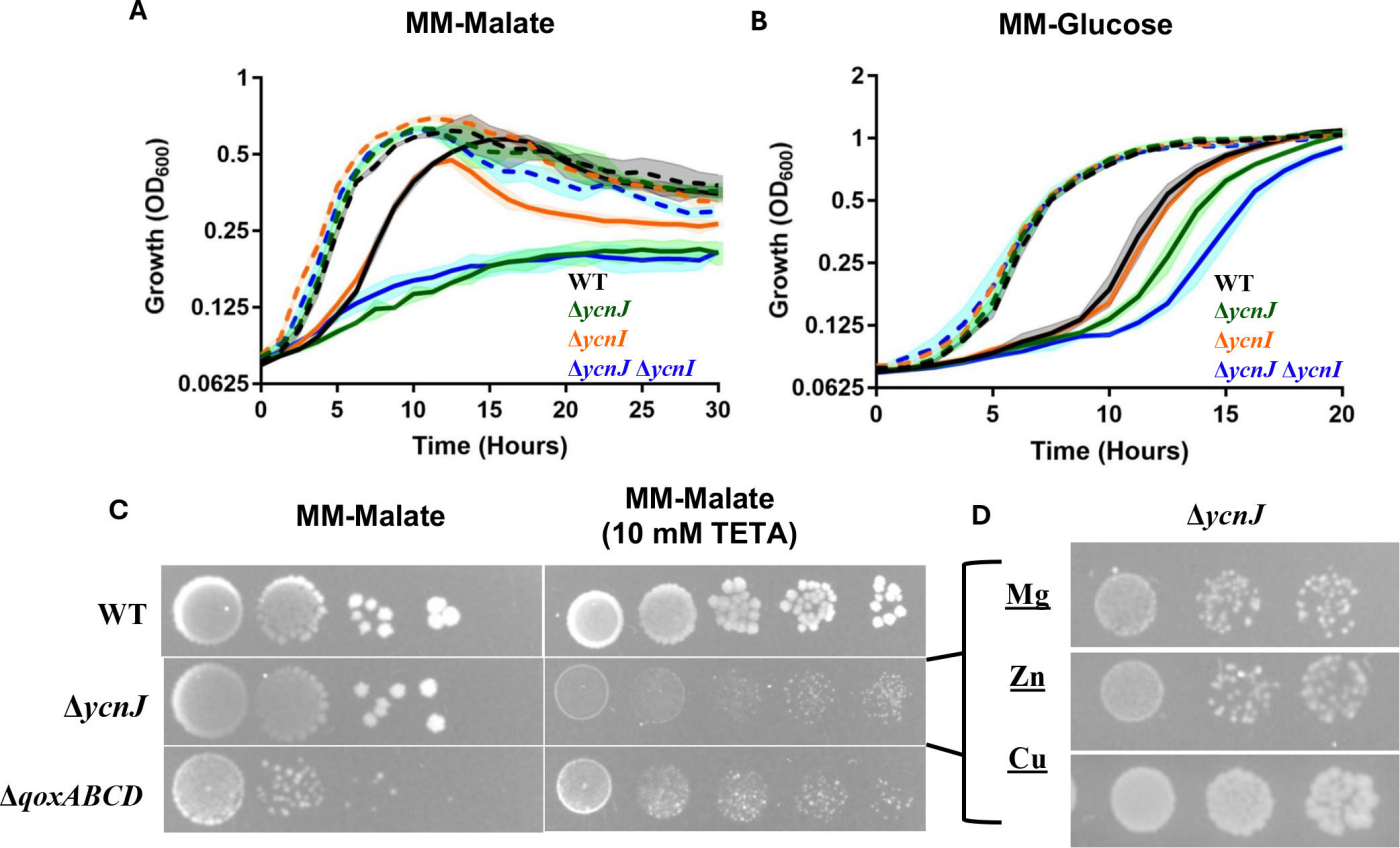
Sensitivity of Δ*ycnJ* and Δ*ycnI* strains to copper ion depletion. Growth curves of WT, Δ*ycnJ*, ΔycnI, and Δ*ycnJ* Δ*ycnI* in minimal medium without (dashed line) and with 10 mM TETA (solid lines). Growth in either (**A**) malate or (**B**) glucose as the sole carbon source. The panels are based on three biological replicates, with the shaded region representing the standard deviation. (**C**) Growth on agar plates spotted with undiluted, 10^−1^-, 10^−2^-, 10^−3^-, and 10^−4^-fold diluted cultures of WT, Δ*ycnJ,* and Δ*qoxABCD* on MM-malate plates with or without 10 mM TETA. (**D**) Growth on plates with 10^−2^-, 10^−3^-, and 10^−4^-fold diluted culture of Δ*ycnJ* spotted on MM-malate plates with 10 mM of TETA supplemented with 10 μM MgCl_2_, 10 μM ZnCl_2_, or 10 μM CuSO_4_. Photos shown are representatives of three independent biological replicates. The data in panel **A** are consistent with results reported previously (36).

The extracellular domains of both YcnI and YcnJ have similarly high-affinity Cu^2+^-binding sites (36, 37). To create copper-limiting growth conditions, we use the Cu^2+^-selective chelator triethylenetetramine (TETA, *K*_*d*_ ~10^−17^ M) (38). Consistent with our previous results (36), supplementation of MM with malate as a carbon source with 10 mM TETA resulted in a modest reduction in growth rate (but not yield) for both wild-type (WT) and ∆*ycnI* mutant strains, whereas a ∆*ycnJ* strain was severely growth impaired (Fig. 1A). The ∆*ycnJ* strain was also inhibited in growth at 2.5 and 5 mM TETA, levels which did not noticeably inhibit the WT or ∆*ycnI* strains (Fig. S1). These results support the proposal that YcnJ is important for growth under copper-limiting conditions (33), whereas YcnI plays a secondary role (36).

We anticipated that one major role for copper would be to support respiration. Consistent with this expectation, when grown on MM with glucose the growth yield of the ∆*ycnJ* strain was restored to that of WT (Fig. 1B). This is because exponential growth on glucose can proceed by fermentation, unlike malate where growth is dependent on the respiratory chain for energy conservation (39). The ∆*ycnJ* and ∆*ycnI* mutations did not have additive effects in MM-malate with TETA (Fig. 1A), consistent with a model in which YcnI supports copper import by YcnJ (36). However, some additivity was seen in MM-glucose medium with TETA (Fig. 1B), which implies that YcnI may function independently of YcnJ under some conditions. Although growth yield is restored when malate is replaced by glucose, 10 mM TETA supplementation results in a long growth lag (Fig. 1B). This lag suggests that copper-dependent enzymes, presumably cytochrome oxidases, are required for optimal growth with glucose. This lag is increased further with ∆*ycnJ* strains.

### The ∆*ycnJ* growth defect is due to a deficiency in Qox function

We hypothesized that the growth defect of the Δ*ycnJ* strain in the presence of TETA results from insufficient copper loading into Qox. To confirm that these phenotypes resulted from copper starvation, we tested the effects of metal supplementation. On MM-malate plates with TETA, the ∆*ycnJ* mutants displayed a small colony size, which was suppressed by copper addition but not zinc or magnesium (Fig. 1C and D; Fig. S2). The small colony size of the Δ*ycnJ* strain is similar to a Δ*qoxABCD* operon deletion strain (Fig. 1C). However, unlike Δ*ycnJ*, the Δ*qoxABCD* strain formed small colonies both with and without TETA.

**Fig 2:**
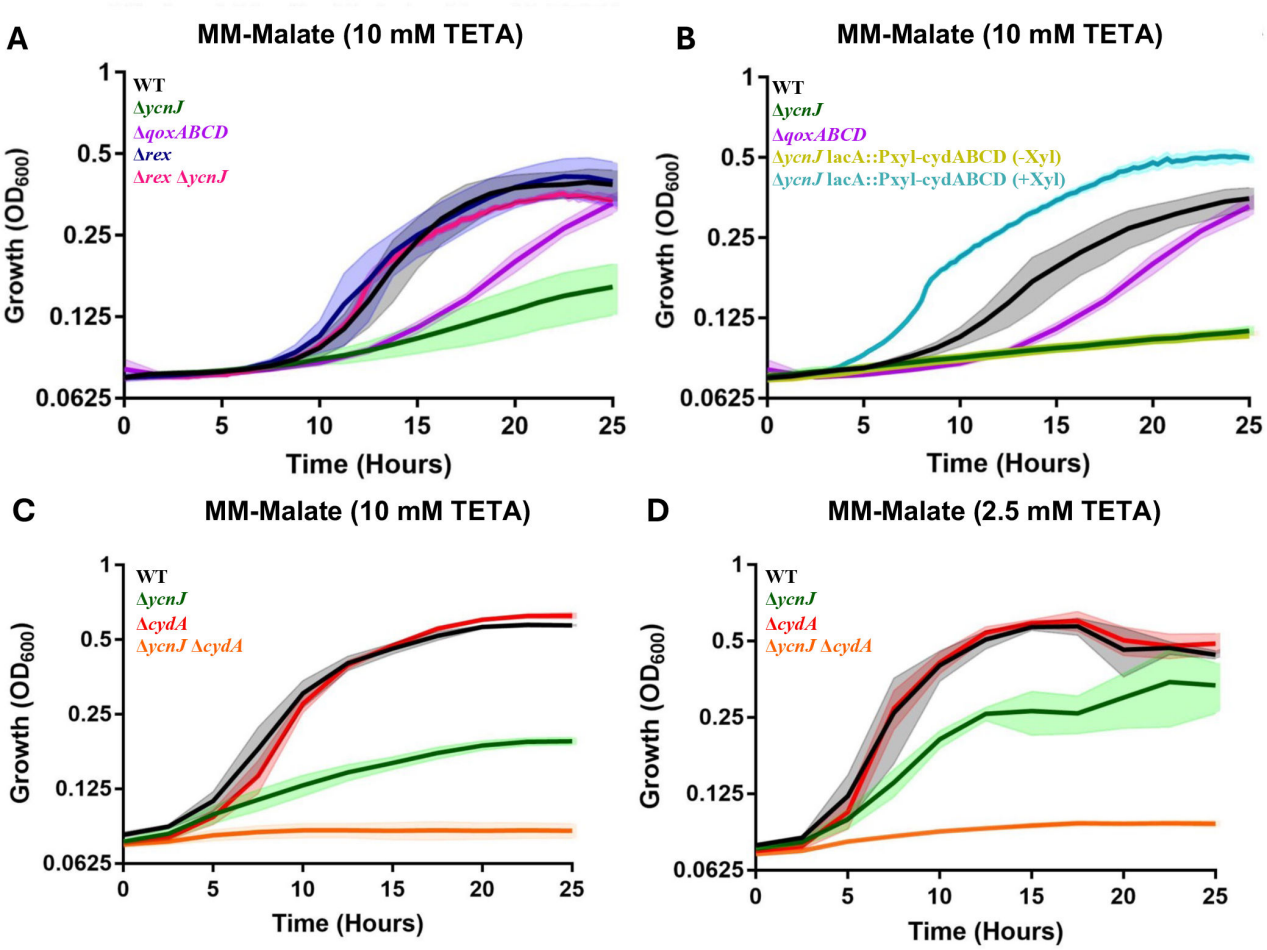
Growth of Δ*ycnJ* strain under copper ion deprivation can be restored through the absence of Rex or overexpression of Cyd. (**A**) WT, Δ*ycnJ*, Δ*rex*, Δ*qoxABCD,* and Δ*rex* Δ*ycnJ* and (**B**) WT, Δ*ycnJ*, Δ*qoxABCD*, Δ*ycnJ* lacA::P_xyl_-*cydABCD* (−Xyl), and Δ*ycnJ lacA*::P_xyl_-*cydABCD* (+Xyl) in MM-malate supplemented with 10 mM TETA. Growth of WT, Δ*ycnJ*, Δ*cydA*, and Δ*ycnJ* Δ*cydA* in MM-malate with (**C**) 10 mM TETA and (**D**) 2.5 mM TETA. Results are from three biological replicates, with the shaded regions representing the standard deviations.

To determine if the small colony phenotype was linked to a deficiency in Qox, we took advantage of the ability of Cyd to functionally complement a Qox-deficient strain (18). Deletion of the *rex* gene, which encodes a repressor of the *cydABCD* operon, restored the growth of Δ*ycnJ* in copper-limited MM-malate (Fig. 2A). Furthermore, expression of the *cydABCD* operon from a xylose-inducible promoter in a Δ*ycnJ* background also restored growth (Fig. 2B). We conclude that the poor growth of Δ*ycnJ* under conditions of copper limitation is due to defective copper loading into Qox, and increased Cyd activity rescues this defect.

Since Cyd is required for growth in the absence of Qox (18), we hypothesized that a Δ*cydA* strain would be more sensitive to copper limitation. Indeed, a ∆*ycnJ* ∆*cydA* strain was highly sensitive to both 10 and 2.5 mM TETA (Fig. 2C and D). In contrast, a Δ*ycnI* Δ*cydA* mutant did not have a growth defect under copper-limiting conditions (Fig. S3). Collectively, these results demonstrate that YcnJ, but not YcnI, is critical for Qox activity under copper-limiting conditions.

### Inactive Cta and Qox are assembled in the membrane under copper deprivation

**Fig. 3:**
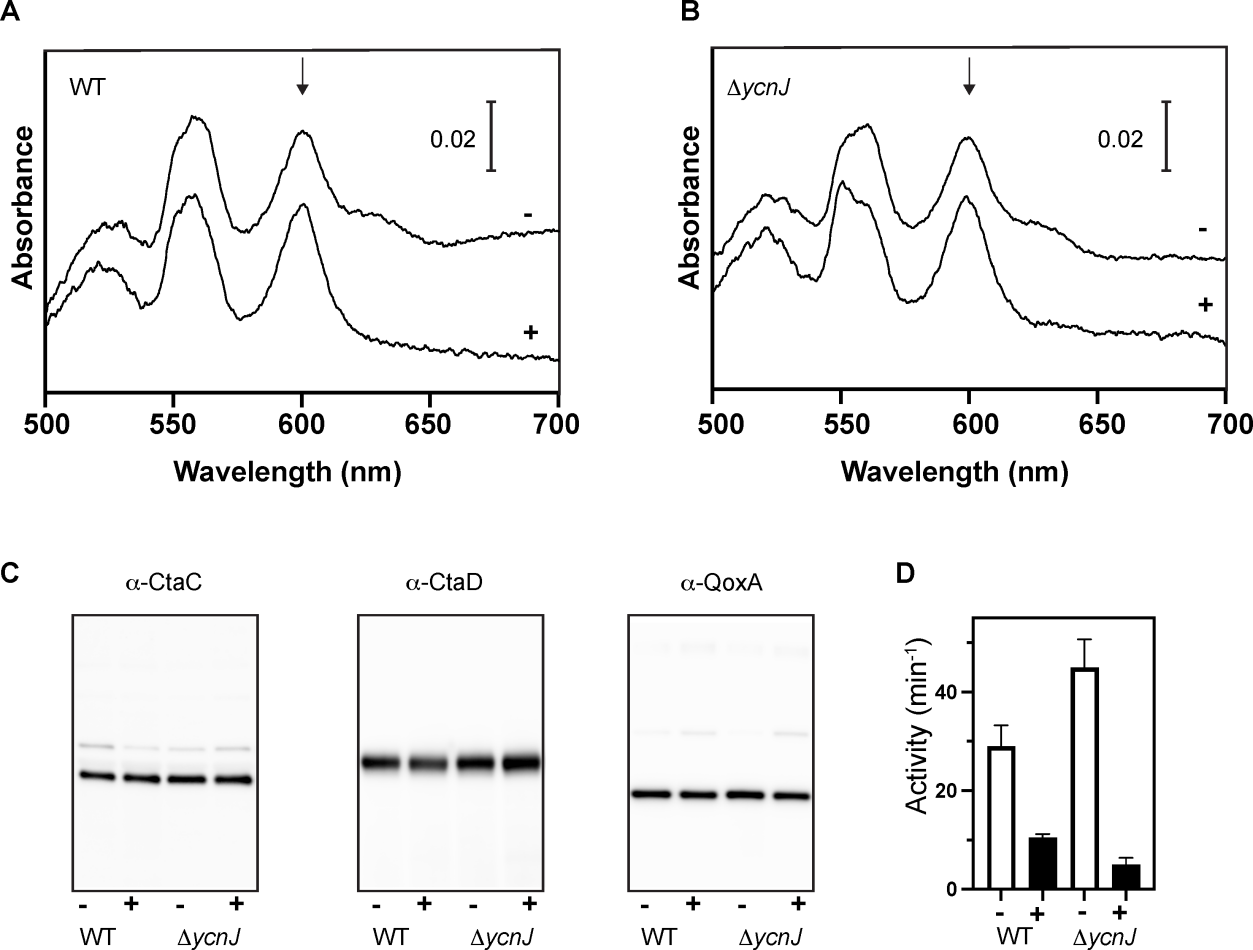
Cytochrome composition, content of CtaC, CtaD, and QoxA, and cytochrome *c* oxidase activity under copper-limiting conditions. Light absorption difference (dithionite-reduced minus ferricyanide-oxidized) spectra of isolated membranes from (**A**) WT (CU1065) and (**B**) Δ*ycnJ* (HB30927). Cells were grown in MM-malate without (−) or with (+) 10 mM TETA (Fig. S7). The vertical bar indicates the absorbance scale, and the arrow marks the ~600 nm absorption maximum of the alpha-band of cytochrome *a* in both Cta and Qox. The protein concentration for the spectra is normalized to 2 mg/mL. (**C**) Immunoblot analysis of membranes from WT and Δ*ycnJ* using antisera directed against subunits CtaC and CtaD of cytochrome *caa*_3_ and QoxA of cytochrome *aa*_3_. Membrane protein (10 μg) was loaded per lane to detect the CtaC, CtaD, and QoxA antigens. (**D**) Cytochrome *c* oxidase activity (turnover number per cytochrome *a*). The error bars represent the standard deviations (*n* = 2). The difference between −TETA and +TETA conditions was significant for both the wild-type and Δ*ycnJ* strains (*P* < 0.05, unpaired *t*-test).

During assembly of Cta, the Cu_A_ center, which is unique for this oxidase, is inserted into the CtaC subunit on the outer side of the cytoplasmic membrane. How the Cu_B_ site is assembled is less clear (5). To biochemically analyze the effects of copper deprivation on Cta and Qox, we grew the WT and ∆*ycnJ* strains in liquid MM-malate with and without 10 mM TETA added. Membranes were isolated and analyzed for the presence of cytochrome *a*, Cta and Qox protein subunits, and cytochrome *c* oxidase activity (Fig. 3). Growth curves showing the time points of TETA addition to the cultures and cell harvest are displayed in Fig. S4. The level of cytochrome *a* (Cta plus Qox), as determined from the absorption peak at 600 nm in reduced membranes, was not decreased by TETA in the growth medium. Similarly, the amounts of CtaC, CtaD, and QoxA polypeptides were largely unaffected by TETA, as determined by immunoblot with antisera specific for these oxidase subunits. In contrast, cytochrome *c* oxidase (Cta) activity was decreased by 65%–85% (Fig. 3D). We infer from these results that, under copper deprivation, Cta and Qox are assembled in the membrane with the heme A groups incorporated. However, the two oxidases assembled under these conditions may be largely inactive, presumably because they lack copper prosthetic groups.

### Both the CopC and CopD domains of YcnJ support Qox function under copper limitation

**Fig 4:**
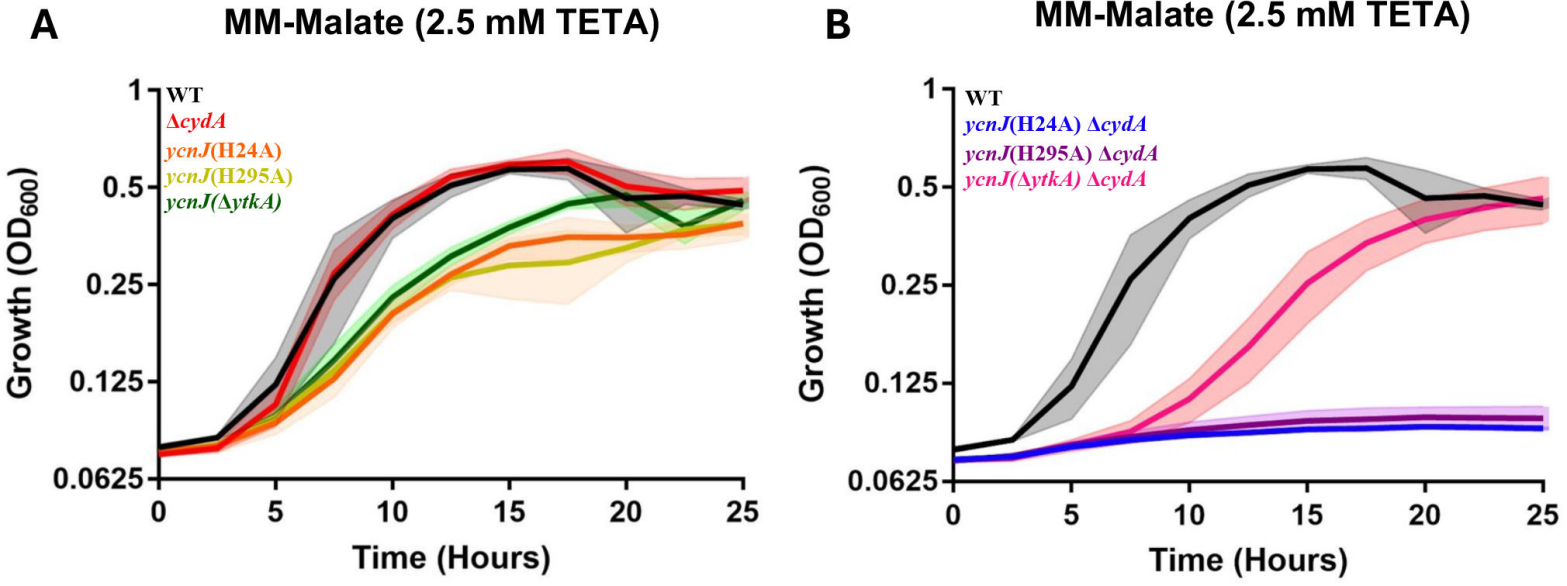
Cu import by YcnJ is required for Qox function under copper-limiting conditions. Growth of (**A**) WT, Δ*cydA*, *ycnJ*(H24A), *ycnJ*(H295A), and *ycnJ*(Δ*ytkA*) and (**B**) WT, *ycnJ*(H24A) Δ*cydA*, *ycnJ*(H295A) Δ*cydA*, and *ycnJ*(Δ*ytkA*) Δ*cydA* in MM-malate with 2.5 mM TETA. Results are from three biological replicates, with the shaded regions representing the standard deviations. Note that some lines have been repeated for clarity.

YcnJ is composed of three domains: CopC, CopD, and YtkA. Most CopC family proteins, including the CopC domain of YcnJ, directly interact with copper (36, 40, 41). As originally described in *Pseudomonas syringae*, periplasmic CopC proteins may function together with transmembrane CopD proteins for copper import (42). In some organisms, CopC and CopD domains are fused into a single polypeptide, as exemplified by *B. subtilis* YcnJ. The role of YcnJ in growth under copper limitation requires a Cu^2+^-binding site in the extracellular CopC domain, which can exchange Cu^2+^ with a structurally similar binding site in YcnI (36). Building on our previous findings, a *ycnJ*(H24A) mutant, deficient in Cu^2+^ binding to the YcnJ CopC domain (36), grew poorly under copper limitation relative to WT (Fig. 4A). This effect is even more dramatic in a *ycnJ*(H24A) ∆*cydA* double mutant (Fig. 4B). The growth defects resulting from the *ycnJ*(H24A) mutation are comparable to those for ∆*ycnJ* (Fig. 2D). This suggests that binding of Cu^2+^ to the extracellular CopC domain of YcnJ is essential for its function.

To determine if the transmembrane CopD domain of YcnJ also supports Qox function, we generated a histidine to alanine point mutation at position 295 (H295A). In the *E. coli* YcnJ homolog YebZ, this conserved histidine residue is important for copper uptake (43). When grown in MM-malate under copper-limiting conditions, the *ycnJ*(H295A) mutant phenocopied Δ*ycnJ* (Fig. 4; Fig. S5). This suggests that under copper starvation, the CopD domain is also required for the function of YcnJ, potentially by mediating copper import and/or metalation of Qox. Since copper-binding residues in both the CopC and CopD domains are required for YcnJ function, we infer that Cu^2+^ cannot bypass the CopC domain and bind directly to the CopD domain under these conditions.

The C-terminal YtkA-like domain of YcnJ is predicted to have structural similarity with CtaK (previously known as YtkA) (15). To test the role of the YtkA domain, we generated a *ycnJ*(∆*ytkA*) mutation by introducing a frameshift mutation after codon 432 (P433stop). Cells expressing YcnJ(∆YtkA) retained the ability to support Qox function, as judged by the ability to support growth in the absence of Cyd. Specifically, in the ∆*cydA* background, *ycnJ*(∆*ytkA*) grew much better in the presence of 2.5 mM TETA (Fig. 4B) than the ∆*cydA* ∆*ycnJ* (Fig. 2D), ∆*cydA ycnJ*(H24A) (mutated CopC), and ∆*cydA ycnJ*(H295A) (mutated CopD) double mutants (Fig. 4B). All strains showed normal growth in the absence of TETA (Fig. S6). Collectively, these results demonstrate that YcnJ functions in support of Qox under conditions of copper limitation and that this role requires the CopC and CopD domains, but not the YtkA domain.

### All three YcnJ domains support Cta function

Deletion of the structural genes for cytochrome *caa*_3_ (*ctaCDEF*) has little effect on growth, and unlike Cyd, Cta is not required for growth in strains lacking Qox (18). Cta activity can be assayed by staining colonies on agar plates with TMPD, a reductant that transfers one electron to the cytochrome *c* of Cta resulting in a blue TMPD cation (44, 45). Using this assay, genes required for Cta function have been identified, including *ctaG*, *ctaK*, and *ctaM* (14, 15).

Here, we have used TMPD staining to explore the role of YcnJ in Cta function. A ∆*ycnJ* strain showed normal TMPD staining, suggesting that YcnJ is not essential for Cta function on MM-glucose plates (with no TETA) or on TBAB plates (Table 1) (Fig. S7). Therefore, we instead tested the effect of *ycnJ* mutations in strains already compromised in Cta function due to a *ctaK* mutation (15). CtaK is a membrane-tethered extra-cytoplasmic lipoprotein thought to function in concert with Sco, a thioredoxin-like copper-binding protein (46). *B. subtilis* Sco can bind either Cu^2+^ or Cu^+^ and is postulated to function in assembly of the Cu_A_ site in Cta (47, 48). As seen previously (15), the TMPD staining defect in strains lacking Sco or CtaK can be rescued by supplementation of the growth medium with copper ions (Table 1). Compared to ∆*ctaK*, a ∆*ycnJ ∆ctaK* double mutant required higher levels of supplemental copper to rescue the TMPD staining defect. This same additivity was seen in the *ycnJ*(H24A) *∆ctaK* and *ycnJ*(H295A) Δ*ctaK* strains (Table 1). We conclude that the role of YcnJ in supporting Cta function likely involves copper-binding to the CopC and CopD domains, presumably as part of a copper import pathway.

**TABLE 1:** Cytochrome *c* oxidase activity of colonies depends on copper ions in the plate^*a*^

CuSO_4_ added (µM)	WT	Δ*sco*	Δ*ctaK*	Δ*ycnJ* Δ*ctaK*	*ycnJ*(Δ*ytkA*) Δ*ctaK*	*ycnJ*(H24A) Δ*ctaK*	*ycnJ*(H295A) Δ*ctaK*	Δ*ycnK* Δ*ctaK*
0	+++	−	−	−	−	−	−	−
0.1	+++	−	−	−	−	−	−	+
0.2	+++	−	+	−	−	−	−	+
0.4	+++	−	+	−	−	−	−	+
0.6	+++	−	++	−	+	−	−	++
0.8	+++	−	++	+	+	+	+	++
1	+++	+	++	+	++	+	+	++
3	+++	++	++	++	++	++	++	++
5	+++	+++	+++	+++	+++	+++	+++	+++

^*a*^ The chart presents the relative TMPD oxidation activity of different *B. subtilis* strains with the indicated mutations spotted on MM-glucose plates supplemented with Cu. Δ*sco* is used as a control, and −, +, ++, and +++ represent no, low, intermediate, and WT staining intensity, respectively. Shading highlights all cells with a positive signal. Strains Δ*ycnK*, Δ*ycnJ*,* ycnJ*(Δ*ytkA*), *ycnJ* (H24A), and *ycnJ* (H295A) behaved like WT and are not shown in the table. This table summarizes results from three biological replicates and was additionally confirmed using tryptose blood agar base agar plates as before (15) (Fig. S7).

As reported previously (15), TMPD staining in ∆*ctaK* is restored at lower concentrations of copper than needed for ∆*sco* (Table 1). Because YcnJ and CtaK both include YtkA domains, we hypothesized that they may be functionally redundant in supporting assembly of the Cu_A_ center in Cta. This redundancy involves the YtkA domain of YcnJ since the *ycnJ*(∆*ytkA*) Δ*ctaK* mutant is almost, but not quite, as copper dependent as Δ*ycnJ* Δ*ctaK* strain (Table 1). This small difference may be due to the ability of the truncated protein to still import copper.

To further investigate the role of YcnJ in supporting Cta activity, we constructed a strain lacking *ycnK*, which encodes a Cu-dependent repressor of the *ycnKJI* operon (33, 34). We predicted that YcnJ levels in the Δ*ycnK* Δ*ctaK* strain might be elevated to compensate for the loss of CtaK. Indeed, copper supplementation restored TMPD staining to the Δ*ycnK* Δ*ctaK* strain at lower concentration than needed for Δ*ctaK* alone (Table 1). We conclude that YcnJ functions in supporting Cta function (as revealed in a ∆*ctaK* mutant background) and this role requires all three domains in YcnJ (CopC, CopD, and YtkA).

### CtaM protects cells from copper overload

The assembly of heme-copper oxidases has been proposed to involve both the import of copper into the cytosol and the export of copper by dedicated export proteins (11). In some organisms, P-type ATPases of the well-studied CopA family (49) serve as copper exporters to support the function of heme-copper oxidases (50, 51). *B. subtilis* encodes a single CopA homolog that is expressed together with the CopZ metallochaperone (27, 30). The *copZA* operon is induced by excess copper and has a detoxification function (26, 29). Mutants lacking CopA do not appear to be defective for Qox (a Δ*copA* Δ*cydA* mutant is viable) or Cta (TMPD staining is normal) (Table S3). These results suggest that there is another copper exporter that supports the function of heme-copper oxidases.

**Fig 5:**
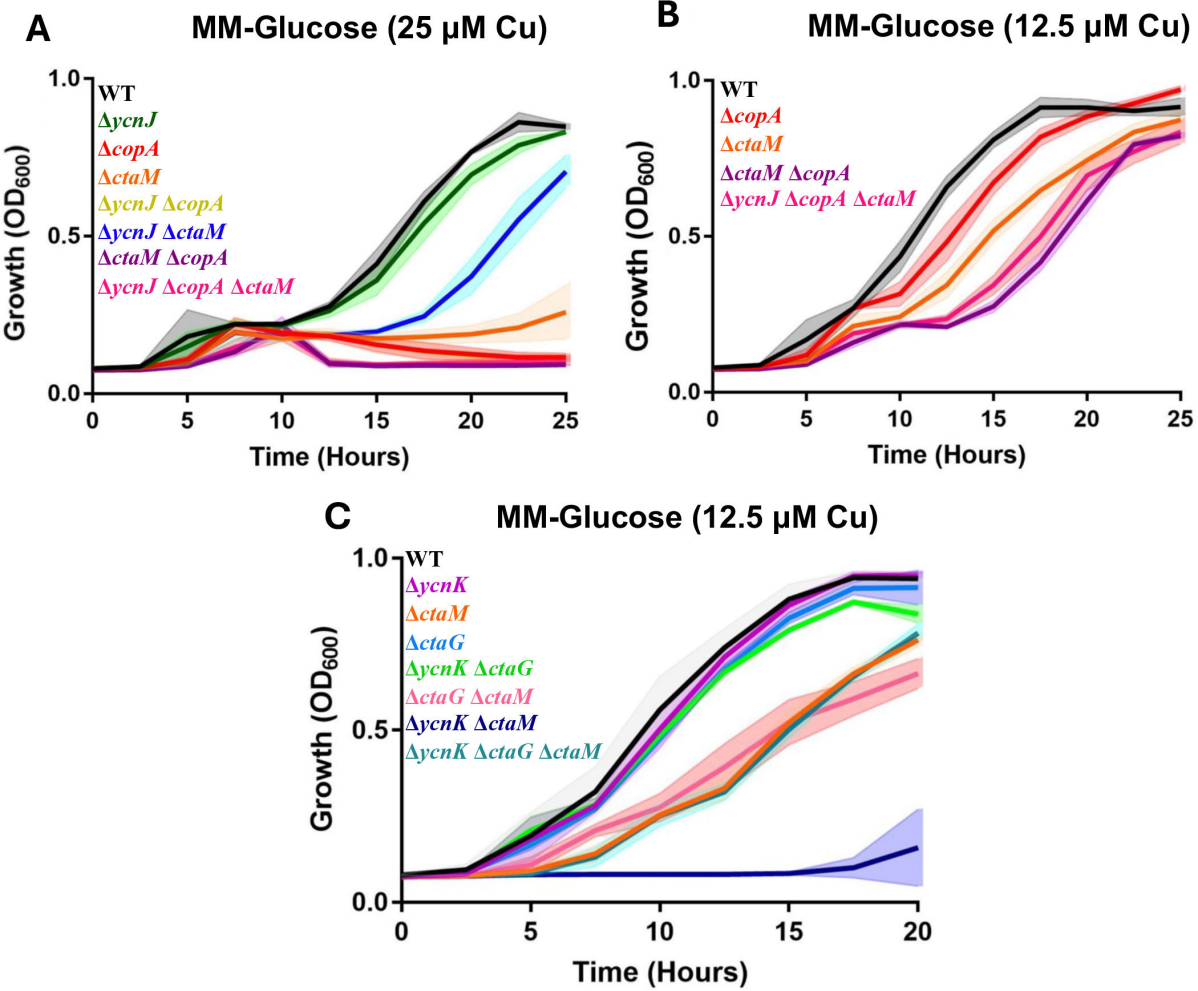
Effects on growth of various mutant strains at elevated copper ion concentration in MM-glucose. (**A**) Growth of WT, Δ*ycnJ*, Δ*copA*, Δ*ctaM*, Δ*ycnJ* Δ*copA*, Δ*ycnJ* Δ*ctaM*, Δ*ctaM* Δ*copA*, and Δ*ycnJ* Δ*copA* Δ*ctaM* in MM-glucose with 25 μM Cu. (**B**) Growth of WT, Δ*copA*, Δ*ctaM*, Δ*ctaM* Δ*copA*, and Δ*ycnJ* Δ*copA* Δ*ctaM* in MM-glucose with 12.5 μM Cu. (**C**) Growth of WT, Δ*ycnK*, Δ*ctaM*, Δ*ctaG*, Δ*ycnK* Δ*ctaG*, Δ*ctaG* Δ*ctaM*, Δ*ycnK* Δ*ctaM*, and Δ*ycnK* Δ*ctaG* Δ*ctaM* in MM-glucose with 12.5 μM Cu. Results are from three biological replicates, with the shaded regions representing the standard deviations.

Previous work has identified several accessory proteins required for heme-copper oxidase function (14, 15). CtaM is a transmembrane protein belonging to the DUF420 family and is required for the assembly of functional Qox and Cta (15, 52). Consistent with its role in Qox function, Δ*ctaM* strains exhibit defective aerobic growth in both MM-glucose and MM-malate (Fig. S8). The poor growth of Δ*ctaM* compared to WT was, as expected, partly suppressed by loss-of-function mutations in *rex* (Fig. S8), which derepresses the copper-independent Cyd (15). To determine if CtaM may function in copper export, we tested a Δ*ctaM* mutant for sensitivity to copper excess. We found that a Δ*ctaM* mutant was sensitive to excess copper, and this phenotype is additive with Δ*copA* (Fig. 5A and B).

We next explored the epistasis between *ctaM* and *ycnJ*. Deletion of *ycnJ* helped reverse the copper sensitivity of ∆*ctaM* but not of ∆*copA* (Fig. 5A). This suggests that CtaM and CopA have physiologically distinct roles and that they may interact with different pools of intracellular copper ions. To further explore the impact of YcnJ on copper sensitivity, we tested ∆*ycnK* strains lacking the repressor of the *ycnKJI* operon. A ∆*ycnK* ∆*ctaM* double mutant had greatly increased copper sensitivity relative to ∆*ctaM* alone (Fig. 5C). In contrast, a ∆*ycnK* ∆*copA* double mutant was only slightly more sensitive to copper than the ∆*copA* single mutant (Fig. S9). These results suggest that in a ∆*ycnK* strain, upregulated for copper import, CtaM (rather than CopA) is most important for copper efflux. We therefore suggest that CtaM works in a pathway of copper trafficking that supports function of heme-copper oxidases, whereas CopA is induced at high levels of Cu^+^ to prevent copper toxicity.

### Functions of CtaG and CtaM in copper trafficking

*B. subtilis* CtaG is a poorly understood transmembrane protein important for the function of Cta but not Qox (14). Importantly, the CtaG protein of *Bacillus* species, as well as other gram-positives such as Actinobacteria, is different from CtaG proteins of gram-negative bacteria, which are orthologs of mitochondrial Cox11. A *B. subtilis* Δ*ctaG* strain retains membrane-localized Cta that contains heme but is inactive (14). Therefore, CtaG may be involved in the assembly of Cu_A_, Cu_B_, or both copper centers. If it is required for the insertion of Cu_B_ into Cta, then other factors perform this function for Qox. To test possible interdependence between CtaG and YcnJ, a Δ*ycnJ* Δ*ctaG* double mutant was constructed and grown in the presence of TETA. Under copper-limiting conditions, Δ*ctaG* showed no growth defect, suggesting that copper import by YcnJ does not require CtaG, and a Δ*ycnJ* Δ*ctaG* mutant phenocopies Δ*ycnJ* (Fig. S10). Thus, YcnJ can function independently of CtaG under copper limitation. Notably, the lack of cytochrome *c* oxidase activity in CtaG-defective mutants is not suppressed by supplementation of the growth medium with copper ions (14).

Next, we explored the interactions of YcnJ, CtaM, and CtaG during growth under copper excess. As noted above, ∆*ctaM* is sensitive to excess copper, and this sensitivity is reduced if YcnJ is absent (Fig. 5A) and enhanced if YcnJ is upregulated (Fig. 5C). For example, the ∆*ctaM* Δ*ycnK* strain displays nearly complete growth inhibition with 12.5 µM copper ions. Remarkably, this inhibition is suppressed when *ctaG* is additionally deleted (Fig. 5C). Interestingly, the intermediate level of sensitivity seen with ∆*ctaM* alone was not suppressed in the ∆*ctaM* ∆*ctaG* double mutant (Fig. 5C). Collectively, these findings suggest that loss of the YcnK regulator sensitizes cells to copper intoxication, and that CtaG enhances copper intoxication resulting from upregulation of YcnJ in cells lacking CtaM.

## DISCUSSION

*B. subtilis* has a branched respiratory chain with three terminal oxidases (the copper-dependent oxidases Qox and Cta, and the copper-independent oxidase Cyd) that support aerobic growth (13, 18). Here, we employ a combination of physiological, genetic, and biochemical assays to investigate proteins that are known or suggested to affect copper trafficking and are implicated in support of Cta or Qox function.

Prior studies suggested that YcnJ supports the growth of *B. subtilis* under copper-limiting conditions generated using bathocuproine disulfonate, a Cu⁺-specific chelator (33). In our studies, we used TETA, a strong Cu^2+^ chelator that acts to sequester adventitious copper present in the growth medium (36). In our minimal growth medium with malate, YcnJ is critical for growth in the presence of 10 mM TETA. This function requires the extracellular CopC domain of YcnJ that chelates Cu^2+^ with a histidine brace motif, consistent with our prior results (36), as well as a predicted copper-binding residue in the transmembrane CopD domain. This supports a model in which the CopD domain receives Cu^2+^ from the CopC domain during copper import. This intramolecular transfer of copper ions on the outer side of the cytoplasmic membrane appears to be obligatory under copper-limiting conditions. However, at higher levels of ambient copper, it remains possible that Cu^2+^ may bypass the CopC domain and bind directly to the CopD domain. A primary role of YcnJ under copper-limiting growth is to support Qox, the major respiratory heme-copper oxidase, as evidenced by the severe exacerbation of growth defects when the alternative, copper-independent Cyd oxidase is absent.

In addition to supporting Qox function, YcnJ also contributes to the function of cytochrome *c* oxidase (Cta). The C-terminal YtkA domain of YcnJ is in the same protein family as CtaK (formerly known as YtkA), which is postulated to function with Sco in the assembly of the Cu_A_ site in Cta (15). By monitoring the ability of supplemental Cu^2+^ to restore Cta activity (TMPD staining), we provide evidence in support of a partially redundant function of YcnJ and CtaK (Table 1). This supports a model in which either YcnJ or CtaK can work in concert with Sco for assembly of Cu_A_. Removal of the YtkA domain from YcnJ also impairs growth under copper limitation (Fig. 4A), although in a ∆*cyd* background this effect is much less dramatic than for either of the Cu-binding mutations in the CopD or CopC domains (Fig. 4B). These findings suggest that the YtkA domain of YcnJ is not essential for Qox function under these conditions (assayed by growth in the ∆*cydA* strain).

The observed role of the transmembrane YcnJ CopD domain in supporting heme-copper oxidase function is consistent with the consensus model for copper trafficking with the transit of copper ions through the cytosol (11). Indeed, Cu^+^ in the cytosol is needed for appropriate regulation of the *copZA* efflux operon (controlled by CsoR [29]) and is proposed to antagonize the YcnK repressor protein (34). Since the heme-copper respiratory oxidases are membrane proteins, and Sco and CtaK are active on the outer side of the cytoplasmic membrane, it is generally assumed that imported copper is subsequently exported from the cell to support assembly of cytochrome oxidases (11).

Some bacteria contain multiple paralogs of the CopA P-type ATPase that can function in copper efflux for either detoxification or assembly of heme-copper oxidases (50, 51, 53). In *B. subtilis*, there is only one CopA P-type ATPase, and it is not required for either Qox or Cta function. It was originally suggested that CtaM may function in heme A biogenesis (52). Qox and Cta proteins with heme A are, however, present in the membrane of Δ*ctaM* cells (15). Thus, we propose that CtaM functions as a copper exporter or has a supportive role in such export. Here, we provide evidence that CtaM is beneficial under conditions of copper excess. This role is additive with CopA, and loss of either protein leads to only a modest increase in copper sensitivity. A role in copper efflux is consistent with genome analyses that suggest that some planctomycetes bacteria appear to encode fusion proteins with both a Sco domain and a downstream CtaM-like (DUF420) domain, as noted in the STRING database (54). We suggest that CtaM may export copper that is then bound to Sco for incorporation into Cta.

In further support for the notion that CtaM functions in copper export, the copper-sensitive phenotype of ∆*ctaM* strains is reduced in cells lacking YcnJ and exacerbated in strains lacking the YcnK repressor and therefore likely to overexpress YcnJ (Fig. 5). This suggests that CtaM functions in the export of copper that is imported by YcnJ. In contrast, the copper-sensitive phenotype of ∆*copA* is not altered by changes in YcnJ expression. The additivity of CtaM and CopA in preventing copper intoxication and the ability of ∆*ycnJ* to suppress ∆*ctaM*, but not ∆*copA*, suggests that these two proteins may access different pools of copper ions. For example, CopA may only function with Cu^+^ bound to CopZ. Since YcnJ preferentially binds Cu^2+^ in its CopC domain, and there are no known reductases associated with YcnJ, it is possible that CtaM functions in reduction and/or export of Cu^2+^. Consistently, *B. subtilis* Sco preferentially binds Cu^2+^ (48). In contrast, CopA is a Cu⁺-specific exporter. In this case, detoxification by CopA may be rate-limited by an as-yet-undefined Cu^2+^ reduction event rather than YcnJ-mediated import. It remains unknown whether copper reduction is enzyme-catalyzed or instead occurs via chemical reduction by abundant low-molecular-weight thiols, such as bacillithiol, which may serve as both a Cu^2+^ reductant (55, 56) and as a buffer of intracellular Cu^+^ (57, 58). Enzyme-mediated Cu^2+^ reductase activity in the membrane, rather than the presumably slower, uncatalyzed reaction of Cu^2+^ by low-molecular-weight thiol compounds in the cytoplasm, may be required for efficient loading of copper ions into oxidase subunits and for minimizing copper toxicity. Such a requirement for a localized, dedicated cupric reductase activity in oxidase biogenesis has been discussed by Marckmann et al. in the context of *Rhodobacter capsulatus* CcoG (59), a protein for which no homolog is present in Firmicutes, such as *B. subtilis*.

Whereas CtaM is important for both Qox and Cta function, the CtaG protein functions only in support of Cta (14). It is not established whether CtaG is required for the formation of Cu_A_ or Cu_B_ in Cta in *B. subtilis* (4). To further explore the role of CtaG, we monitored the genetic interactions between *ctaG* and other genes involved in copper homeostasis. A ∆*ctaG* mutant is not strongly affected in growth under TETA-imposed copper limitation, nor is it additive with ∆*ycnJ* (Fig. S10), consistent with the idea that CtaG is not important for Qox function. Unlike ∆*ctaM*, the ∆*ctaG* mutant does not display copper sensitivity (Fig. 5C). Curiously, the high copper sensitivity noted for the Δ*ctaM* Δ*ycnK* strain is suppressed in a Δ*ctaM* Δ*ycnK ∆ctaG* strain. We suggest that the high copper sensitivity of the Δ*ctaM* Δ*ycnK* strain results from a lack of a putative copper efflux function and upregulation of copper import due to the loss of the YcnK repressor protein. The suppression of this phenotype in the absence of CtaG suggests that CtaG may interact with YcnJ, CtaM, or both. Support for a model in which CtaG supports the import function of YcnJ under copper excess is provided by the observation that *Corynebacterium glutamicum* encodes a fusion protein in which CtaG is fused to a CopD domain similar to the CopD domain in YcnJ (60). We therefore speculate that the loss of CtaG in *B. subtilis* may reduce YcnJ-mediated copper import, thereby helping to reduce the copper sensitivity in the Δ*ctaM* Δ*ycnK* strain. Testing this model will require the development of biochemical methods to monitor copper import and export in this system.

**Fig 6:**
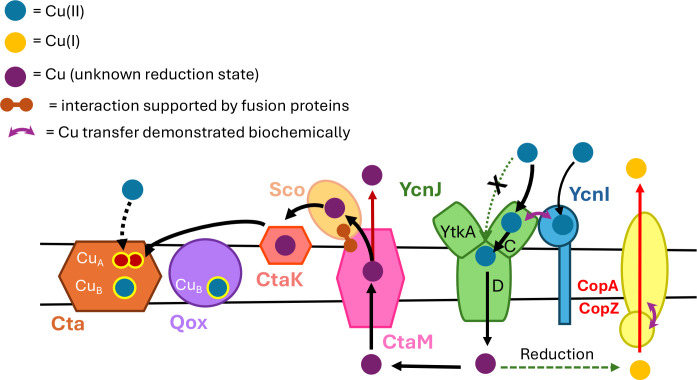
Potential pathways of copper trafficking in *B. subtilis*. Each arrow represents a possible pathway of copper flow in *B. subtilis*, as explored in further detail below. YcnJ is the central protein in Cu^2+^ import and has three domains: CopC (C), CopD (D), and YtkA (homologous to CtaK). The black arrows represent the major flow of Cu^2+^ through the cell on the path to metalate Qox or Cta. Double-headed purple arrows indicate the biochemically demonstrated transfer of Cu between YcnI and YcnJ (36) and between CopZ and CopA (30). Connected circles (deep red) indicate protein-protein interactions inferred from naturally occurring fusion proteins (see text). The “X” and dotted green line indicate that we do not find support for an ability of Cu^2+^ to directly bind to the CopD domain of YcnJ. Cu^2+^ may be directly incorporated into Cta from the environment during assembly of the Cu_A_ site, as supported by the ability of supplemental copper to compensate for the loss of the Sco-dependent copper delivery pathway. Although not illustrated here, the YcnJ(YtkA) domain is overlapping in function with CtaK (see text). The red arrows represent copper efflux of either Cu^+^ (by CopA) or Cu^2+^ (proposed to be exported by CtaM).

In summary, our findings show that YcnJ functions to support the activity of the two heme-copper oxidases, Cta and Qox. YcnJ can work independently or in concert with YcnI to support Qox function under copper-limiting conditions (Fig. 6). Copper-binding residues in both the CopC and CopD domains of YcnJ are important for the function of the heme-copper oxidases, consistent with a role in copper import (Fig. 6). The YtkA domain of YcnJ additionally serves a partially redundant role with CtaK in the assembly of the Cu_A_ site in Cta. Under conditions of copper deprivation, enzymatically inactive heme A-containing Qox and Cta assemble in the membrane, suggesting inadequate copper metalation. The trafficking of copper after import by YcnJ is less clear. Here, we provide suggestive evidence to support a role for CtaM in a copper export (Fig. 6). Further biochemical and structural studies will be required to test and refine this speculative model for copper trafficking.

## MATERIALS AND METHODS

### Bacterial strains and growth conditions

All strains used in this study are referenced in Table S1. Gene deletions were created using the BKE collection obtained from the *Bacillus* Genetic Stock Center (61). Mutations were moved into the desired genetic background and selected on rich medium with proper antibiotics. The rich medium for growth was lysogeny broth (LB) (Affymetrix) with erythromycin (1 μg/mL), lincomycin (25 μg/mL), or kanamycin (15 μg/mL), as needed. Markerless deletions were made with the pDR244 plasmid and confirmed using the check primers listed in Table S2 (61).

Truncation of the YtkA domain of YcnJ and the point mutations in the CopC (H24A) and CopD (H295A) domain were generated using a simplified CRISPR-Cas9 method for *B. subtilis* (62) and the primers listed in Table S2. Repair fragments were amplified and stitched using long-flanking homology PCR. The upstream and downstream primers were designed with flanking SfiI sites for downstream digestion, and the central primers contained the stop codon. Stitched products and the pAJS23 plasmid were digested with SfiI (New England Biolabs [NEB]) at 50°C for 2 h. The pAJS23 plasmid contained the guide RNA against the *erm* cassette. The digested stitched products were purified (OMEGA) and ligated into the pAJS23 through T4 DNA ligase (NEB). Ligated product was transformed into *E. coli* DH5α and selected on LB agar plates supplemented with 30 μg/mL of kanamycin. Plasmid was isolated from the clones and confirmed by Sanger sequencing (BRC at Cornell). Confirmed plasmid was transformed into *E. coli* TGI. Plasmid was isolated from TGI and moved into *ycnJ::erm* recipient strain. Recipient strain was grown in 5 mL of modified competence medium to an OD_600_ of ~0.8 before 1 μg plasmid was added. The culture was then incubated at 30°C for 2 h and then plated on LB agar with 15 μg/mL kanamycin plus 0.2% mannose. Plates were incubated at 30°C for 48 h. To remove the CRISPR plasmid, transformants were repeatedly patched on LB agar for 3 days at 45°C. Clones sensitive to kanamycin and erythromycin were selected and confirmed by Sanger sequencing.

Chemically defined MM contained 10 g/L ammonium sulfate (NH_4_)_2_SO_4_, 5 g/L trisodium citrate (Na_3_C_6_H_5_O_7_•2H_2_O), 5 g/L l-glutamic acid (potassium salt monohydrate), 40 mM MOPS (pH 7.4), 2 mM KPO_4_ (pH 7.0), 10 mg/L tryptophan, 0.8 mM MgSO_4_, 50 μM ferric ammonium citrate, 5 μM MnCl_2_, and either 0.8% (wt/vol) malate (pH 7.4) (MM-malate) or 2% (wt/vol) glucose (MM-glucose) as a carbon source. In Cu-depleted conditions, 10 mM of TETA (Sigma Chem. Co.) was added to the MM-malate medium. For xylose-inducible strains, 1% (wt/vol) xylose was added to the growth medium.

Copper ion limitation by TETA was confirmed by spot dilution on MM-malate plates. The plates were prepared by mixing 2× strength MM-malate with 3% agar. MM-malate plates were then supplemented with 10 mM TETA and either 10 μM ZnCl_2_, 10 μM MgCl_2_, or 10 μM CuSO_4_.

For spot dilution, cultures were grown on LB agar overnight at 37°C. Cells were transferred to 5 mL of LB and grown until OD_600_ reached ~0.4. These cultures were washed one time with MM-malate and then serially diluted in MM-malate in a microtiter plate. Diluted culture (5 μL) was spotted, and plates were allowed to dry for 15 min in a laminar hood. Plates were then incubated overnight at 37°C.

For spread plating, cultures were grown on LB agar overnight at 37°C. Cells were then transferred to 5 mL of LB and grown until the OD_600_ reached ~0.4. These cultures were washed one time with MM-malate and then serially diluted in MM-malate in microcentrifuge tubes. One hundred microliters of 10^−5^ dilution was spread with glass beads on the plates and allowed to dry for 15 min in a laminar hood. Plates were then incubated for 40 h at 37°C.

### Growth measurements

Bacterial cultures were streaked from frozen glycerol stocks on LB agar plates overnight at 37°C. Cells were transferred to 5 mL of LB and were aerobically grown at 37°C until the OD_600_ reached ~0.4. From these mid-log cultures, 1 mL was transferred to a microcentrifuge tube and washed with MM-malate or MM-glucose, respectively. In a 100-well Bioscreen C honeycomb microplate (Steri), 200 μL of MM-malate or MM-glucose was dispensed, and to this, 2 μL of bacterial inoculum was added. Growth was monitored periodically for 24–48 h with shaking at 37°C using a Bioscreen C growth curve analyzer.

### TMPD staining

Cytochrome *c* oxidase activity was measured using colony overlay staining with TMPD essentially as before (45). For staining of colonies on MM-glucose plates, strains were grown from frozen glycerol stocks on LB agar plates overnight at 37°C. Cells were then transferred to 5 mL of LB and were aerobically grown at 37°C until the OD_600_ reached ~0.4. One mL of cells was then transferred to a microcentrifuge tube and washed with MM-glucose. Five microliters of washed cells was then spotted on MM-glucose plates supplemented with 0, 0.1, 0.2, 0.4, 0.6, 0.8, 1, 3, or 5 μM CuSO_4_. After incubation of plates overnight at 37°C, they were frozen at −20°C for 20 min and then thawed for 15 min in a laminar hood. Soft agar containing 0.8 mL of 10% (wt/vol) Triton X-100 in 0.1 M potassium phosphate buffer (pH 7.0), 0.2 mL of 10% (wt/vol) sodium deoxycholate, 1 mL of 100% ethanol, 1 mL of 1% TMPD, and 2 mL of 1.5% melted soft agar was poured on top of the plates. TMPD oxidase activity (blue coloration of colonies) was registered within 5–15 min.

### Isolation of membranes and biochemical analyses

To isolate the membrane fraction, cells were grown overnight at 30°C on MM agar (1.5%) plates with glucose (2%) or L-malate (0.8%). Cells from five plates were resuspended in 15 mL MM with the appropriate carbon source. The cultures were grown for 30 min in 250 mL baffled E-flasks at 37°C at 200 rpm and used to inoculate two 1 L cultures in 5 L baffled E-flasks to an OD_600_ of 0.075. TETA to a final concentration of 10 mM was added when the cultures entered the exponential growth phase (Fig. S4). Membranes were isolated as described previously (63) and suspended in 20 mM sodium 3-(N-morpholino)propanesulfonic acid (pH 7.4) and snap frozen in liquid nitrogen and stored at −80°C until used. Protein concentration was determined using the BCA assay (Pierce) with bovine serum albumin as the standard. Light absorbance spectroscopy at room temperature and cytochrome *c* oxidase activity assays using reduced horse heart cytochrome *c* were essentially done as described before (14). Immunoblot analysis was done as described before (15).

## SUPPLEMENTAL MATERIAL

Supplemental Material mbio.01069-26-s0001.docx Tables S1 to S3; Figures S1 to S10.

ASM does not own the copyrights to Supplemental Material that may be linked to, or accessed through, an article. The authors have granted ASM a non-exclusive, world-wide license to publish the Supplemental Material files. Please contact the corresponding author directly for reuse.
