# Supplementary material for: Pathways of copper import and utilization that support respiration in Bacillus subtilis
Source: mBio. 2026 Jun 9;17(7):e01069-26. doi: 10.1128/mbio.01069-26 (PMC13344008; doi:10.1128/mbio.01069-26)
Supplement: Supplemental Material — Tables S1 to S3; Figures S1 to S10. [file mbio.01069-26-s0001.docx]

**SUPPLEMENTARY MARTERIAL**

**Pathways of copper import and utilization that support respiration in *Bacillus subtilis***

Grayson Barnes^a^, Lars Hederstedt^b^, Oriana S. Fisher^c^, Claes von Wachenfeldt^b^,

and John D. Helmann^a^

^a^Department of Microbiology, Cornell University, Ithaca, NY 14853-8101, USA

^b^The Microbiology Group, Department of Biology, Lund University, Lund, Sweden.

^c^Department of Molecular Biology and Biochemistry, Wesleyan University, 52 Lawn Ave, Middletown, CT USA 06457

**Table S1: Strain List**

| **Strain** | **Genotype** | **Construction** | **Reference** |
| --- | --- | --- | --- |
| 1A1 | 168 WT (*trp*C2) | Lab strain | BGSC |
| CU1065 | 168 *trp*C2 *att*SPβ | Lab strain | BGSC 1A100 |
| HB30921 | Δ*ycnJ*::*erm* | BGSC gDNA→CU1065 | This work |
| HB30922 | Δ*ycnI*::*erm* | BGSC gDNA→CU1065 | This work |
| HB30927 | Δ*ycnJ* | pDR244→HB30921 | This work |
| HB30930 | Δ*ycnI* | pDR244→HB30922 | This work |
| HB31050 | Δ*rex*::*kan* | BGSC gDNA→CU1065 | This work |
| HB31040 | Δ*ycnJ* Δ*rex*::*kan* | HB31050 gDNA→HB30927 | This work |
| HBYL1238 | *lacA*::P_xyl_-*cydABCD* (*erm*) | pAX01-*cydABCD*→CU1065 | (1) |
| HB31074 | Δ*ycnJ* *lacA*::P_xyl_-*cydABCD* (*erm*) | pXyl-*cydABCD*→HB30921 | This work |
| HB31185 | Δ*sco*::*erm* | BGSC gDNA→CU1065 | This work |
| HB31188 | Δ*ctaK*::*erm* | BGSC gDNA→CU1065 | This work |
| HB30964 | *ycnJ* P433stop | CRISPR | This work |
| HB31194 | Δ*ycnJ* Δ*ctaK*::*erm* | HB31188 gDNA→HB30927 | This work |
| HB31197 | *ycnJ* P433stop Δ*ctaK*::*erm* | HB31188 gDNA→HB30964 | This work |
| HB31303 | Δ*ctaM*::*erm* | BGSC gDNA→CU1065 | This work |
| HB31327 | Δ*ycnJ* Δ*ctaM*::*erm* | HB31303 gDNA→HB30927 | This work |
| HB31002 | Δ*ycnJ* Δ*ycnI*::*erm* | LFH PCR | This work |
| HB31306 | Δ*ycnJ* Δ*ycnI* | pDR244→HB31002 | This work |
| HB31297 | Δ*ycnJ* Δ*ctaK* | pDR244→HB31194 | This work |
| HB31164 | Δ*copA*::*erm* | BGSC gDNA→CU1065 | This work |
| HB31328 | Δ*ycnJ* Δ*copA*::*erm* | HB31164 gDNA→HB30927 | This work |
| HB31331 | Δ*copA* | pDR244→HB31164 | This work |
| HB31348 | Δ*copA* Δ*ctaM*::*erm* | HB31303 gDNA→HB31164 | This work |
| HB31334 | Δ*ycnJ* Δ*copA* | pDR244→HB31328 | This work |
| HB31346 | Δ*ycnJ* Δ*copA* Δ*ctaM*::*erm* | HB31303 gDNA→HB31328 | This work |
| HB31374 | Δ*ctaG*::*erm* | BGSC gDNA→CU1065 | This work |
| HB31386 | Δ*ycnJ* Δ*ctaG*::*erm* | HB31374 gDNA→HB30927 | This work |
| HB31383 | Δ*ctaM*::*kan* | BGSC gDNA→CU1065 | This work |
| HB31395 | Δ*ctaM*::*kan* Δ*ctaG*::*erm* | HB31374 gDNA→ HB31383 | This work |
| HB31371 | Δ*ctaM*::*erm* Δ*rex*::*kan* | HB31050 gDNA→ HB31383 | This work |
| HB31403 | *ycnJ* H295A | CRISPR | This work |
| HB30963 | *ycnJ* H24A | CRISPR | (2) |
| HB30920 | Δ*ycnK*::*erm* | BGSC gDNA→CU1065 | This work |
| HB30924 | Δ*ycnK* | pDR244→HB30920 | This work |
| HB31413 | Δ*ycnK* Δ*ctaM*::*kan* | HB31383 gDNA→ HB30924 | This work |
| HB31407 | Δ*cydA*::*kan* | BGSC gDNA→CU1065 | This work |
| HB31434 | *ycnJ* H24A Δ*cydA*::*kan* | HB31407 gDNA→ HB30963 | This work |
| HB31436 | *ycnJ* H295A Δ*cydA*::*kan* | HB31407 gDNA→ HB31403 | This work |
| HB31438 | *ycnJ* P433stop  Δ*cydA*::*kan* | HB31407 gDNA→ HB30964 | This work |
| HB31444 | Δ*ycnK* Δ*copA*::*erm* | HB31164 gDNA→ HB30924 | This work |
| HB31445 | Δ*ycnI*::*erm* Δ*cydA*::*kan* | HB31407 gDNA→ HB30922 | This work |
| HBYL1088 | Δ*qoxABCD*::*erm* | LFH PCR | (1) |
| LUT3 | *trpC2 ctaCD::cat* | Lab stock | (3) |

**Table S2: Primer List**

| **Primer** | **Sequence** | **Reference** |
| --- | --- | --- |
| YcnJ-Check-FP | acttgaccatgccgaacgat | This work |
| YcnJ-Check-RP | accttcgttgtcggcagatt | This work |
| YcnI-Check-FP | ggcgtcttccaagatcaaaacc | This work |
| YcnI-Check-RP | gaggagatcgccgcagtag | This work |
| Rex-Check-FP | gctttcattgagcggggtag | This work |
| Rex-Check-RP | ctccagcagcgacatttgat | This work |
| CtaK-Check-FP | cccttaatatcgtacgctaaaacc | This work |
| CtaK-Check-RP | aagcttcatgatctggaagg | This work |
| RF 1 YcnJ FP | AAGGCCAACGAGGCCagtcccttctgtttcgttttgttgc | This work |
| RF 2 YcnJ RP | AAGGCCTTATTGGCCcagctacgtcacaggcatcacgttat | This work |
| RF1 YcnJ TruncPro433 RP | cgctttggtttggtagaattattccggcgctggctcaggc | This work |
| RF 2 YcnJ TruncPro433 FP | gcctgagccagcgccggaataattctaccaaaccaaagcg | This work |
| CtaM-Check-FP | aacgaatggttattgccggg | This work |
| CtaM-Check-RP | ggaggaacgcatactgagga | This work |
| MLS FP | gaatacgggtttgctaaaag | This work |
| MLS RP | cttttagcaaacccgtattc | This work |
| CopA-Check-FP | tgtcagcactgcgtcaaagc | This work |
| CopA-Check-RP | attgctgcaatccaaaccgtc | This work |
| YcnJ His295Ala RF 1 RP | cagatggaagcggaagttaaagcaaggaaatccatcaaaatgc | This work |
| YcnJ His295Ala RF 2 FP | gcattttgatggatttccttgctttaacttccgcttccatctg | This work |
| YcnJ His24Ala RF 1 RP | tgaagatttgacaatataggcggcagcaaagcttgttttcggcag | (2) |
| YcnJ His24Ala RF 2 FP | ctgccgaaaacaagctttgctgccgcctatattgtcaaatcttca | (2) |
| CydA-Check-FP | ttgccgcggaactcaaac | This work |
| CydA-Check-RP | gtcatttggtcagtctggca | This work |
| YcnJ His295Ala FP Seq | cgatcgcagcattggggctg | This work |
| YcnJ TruncPro433 FP Seq | ccatacagcgtacggacaagc | This work |
| YcnK-Check-FP | gtcaaaggtttgccgcagtt | This work |
| YcnK-Check-RP | attcgctgttttccccaggt | This work |

| **CuSO_4_**  **Added**  **(µM)** | **WT** | **Δ*sco*** | **Δ*ctaK*** | **Δ*copA*** |
| --- | --- | --- | --- | --- |
| **0** | **+++** | **-** | **-** | **+++** |
| **0.1** | **+++** | **-** | **-** | **+++** |
| **0.2** | **+++** | **-** | **+** | **+++** |
| **0.4** | **+++** | **-** | **+** | **+++** |
| **0.6** | **+++** | **-** | **++** | **+++** |
| **0.8** | **+++** | **-** | **++** | **+++** |
| **1** | **+++** | **+** | **++** | **+++** |
| **3** | **+++** | **++** | **++** | **+++** |
| **5** | **+++** | **+++** | **+++** | **+++** |

**Table S3. Δ*copA* has normal TMPD staining.** Chart representing the TMPD oxidation activity of different *B. subtilis* mutants spotted on MM-glucose plates supplemented with Cu. The  -, +, ++, and +++ signs represent no, low, intermediate, and WT staining intensity. Chart is a representation of 3 biological replicates.

SI FIGURES


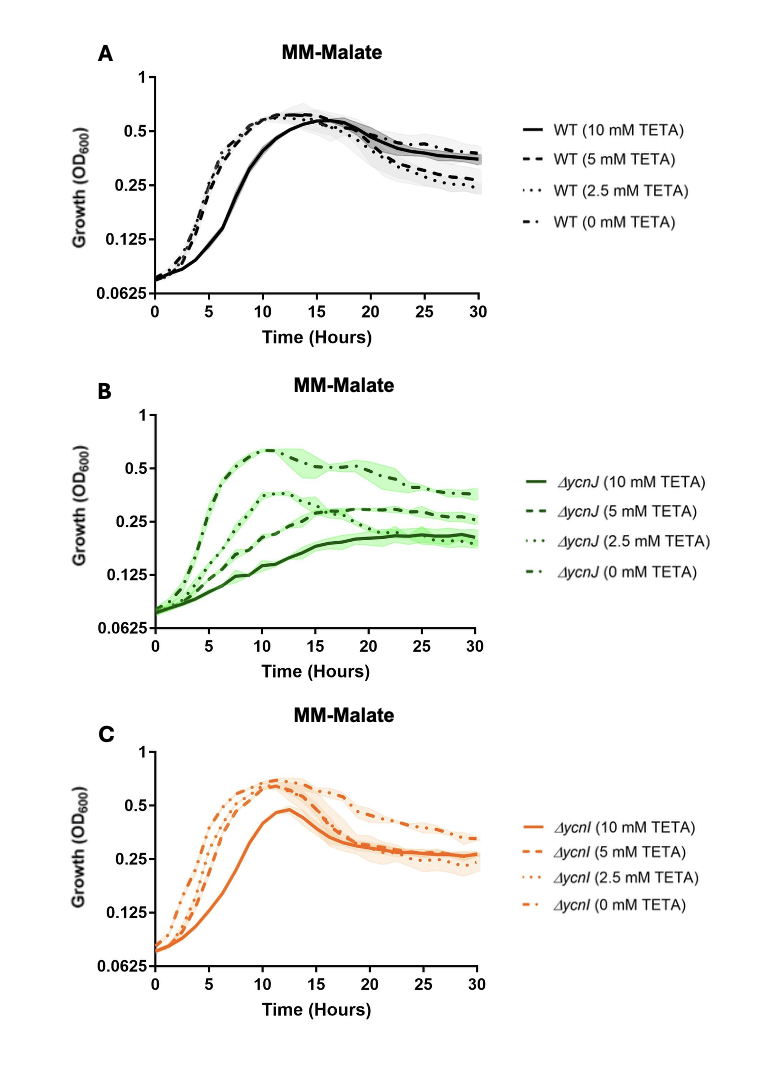


**Supplemental Figure 1. WT, Δ*ycnJ,* and Δ*ycnI* growth at different concentrations of TETA.** Growth of (A) WT, (B) Δ*ycnJ,* and (C) Δ*ycnI* at 10 mM TETA (solid line), 5 mM TETA (dashed line), 2.5 mM TETA (dotted line), and 0 mM TETA (dashed and dot line) in MM-malate. Results are from 3 biological replicates with the shaded region representing the standard deviation.


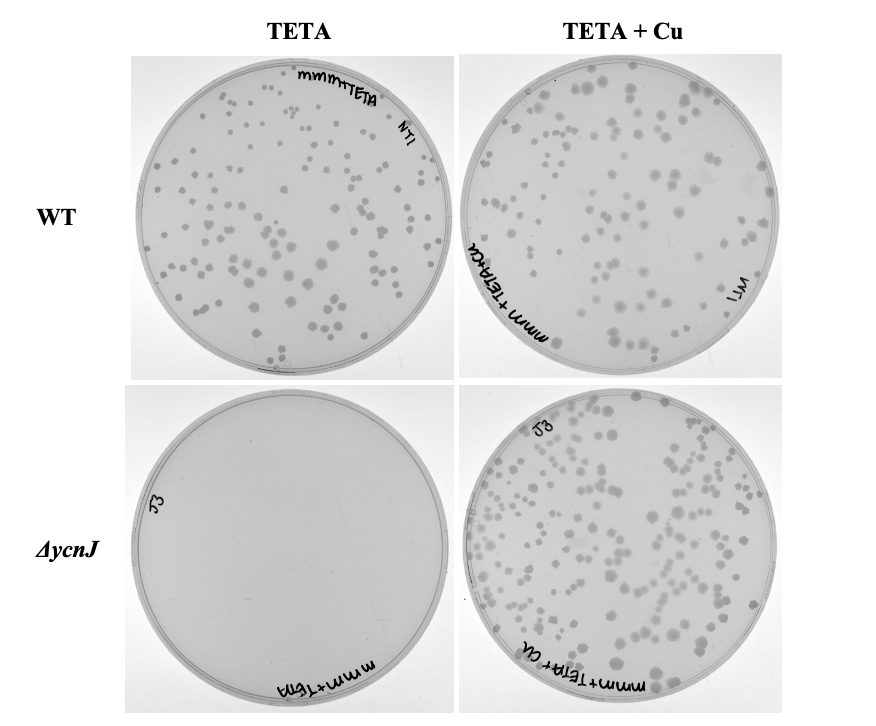


**Supplemental Figure 2. Δ*ycnJ* and WT growth on MM-malate plates with and without Cu supplementation.** Δ*ycnJ* and WT were spread on MM-malate plates with 10 mM TETA. In the case of Cu supplementation, 10 μM Cu was added in addition to the 10 mM TETA. Results are from 3 biological replicates.


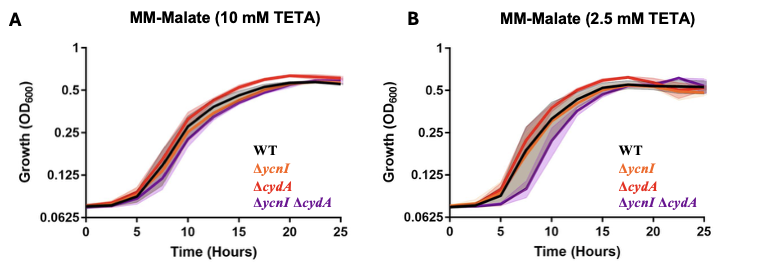


**Supplemental Figure 3. YcnI is not required for the metalation of Qox.** Growth of WT, Δ*ycnI,* Δ*cydA,* and Δ*ycnI* Δ*cydA* in MM-malate supplemented with (A) 10 mM TETA and (B) 2.5 mM TETA. Results are from 3 biological replicates with the shaded region representing the standard deviation.


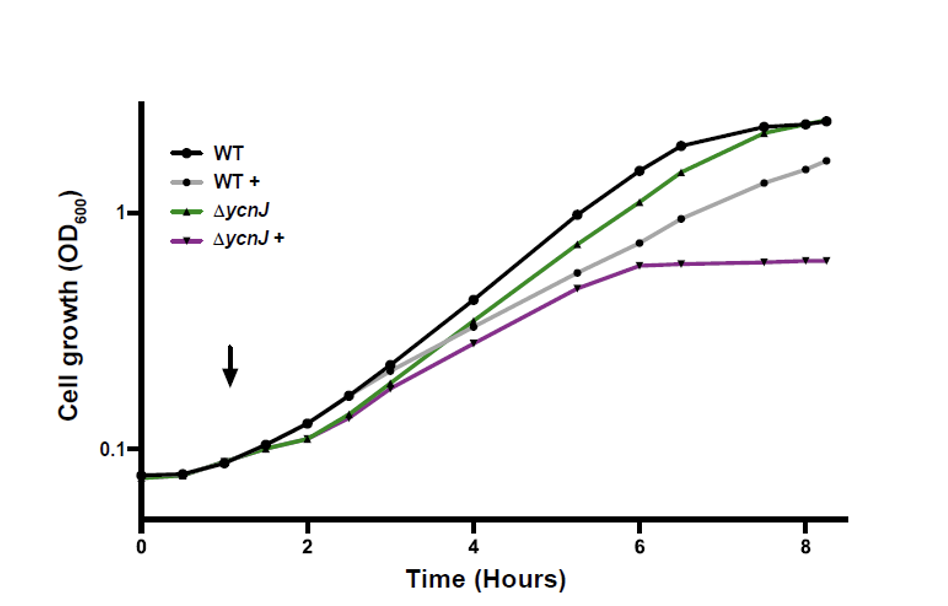


**Supplemental Figure 4.** Growth curves of WT and ∆*ycnJ* strains in MM with and without 10 mM TETA added at early exponential growth phase (arrow). The cells were harvested 8 hrs after inoculum and used for membrane isolation.


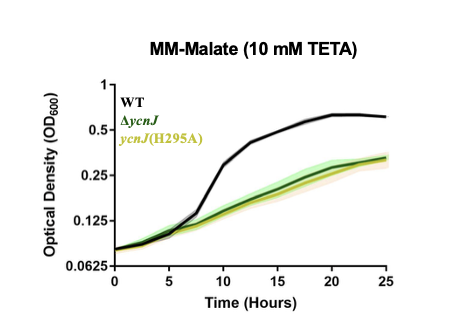


**Supplemental Figure 5. YcnJ^CopD^ is required for the formation of functional Qox.** Growth of WT, Δ*ycnJ,* and *ycnJ*(H295A) in MM-malate supplemented with 10 mM TETA. Results are from 3 biological replicates with the shaded region representing the standard deviation.


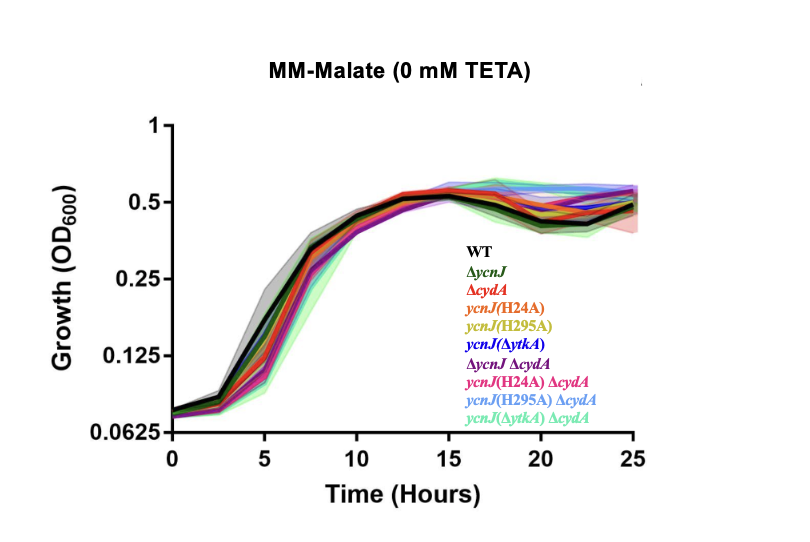


**Supplemental Figure 6*.* No strains in the Δ*cydA* background were found to have a growth defect in MM-malate.** Growth of WT, Δ*ycnJ,* Δ*cydA, ycnJ*(H24A)*, ycnJ*(H295A), *ycnJ*(Δ*ytkA*), Δ*ycnJ* Δ*cydA, ycnJ*(H24A) Δ*cydA, ycnJ*(H295A) Δ*cydA* and *ycnJ*(Δ*ytkA*) Δ*cydA* in MM-malate. Results are from 3 biological replicates with the shaded region representing the standard deviation.


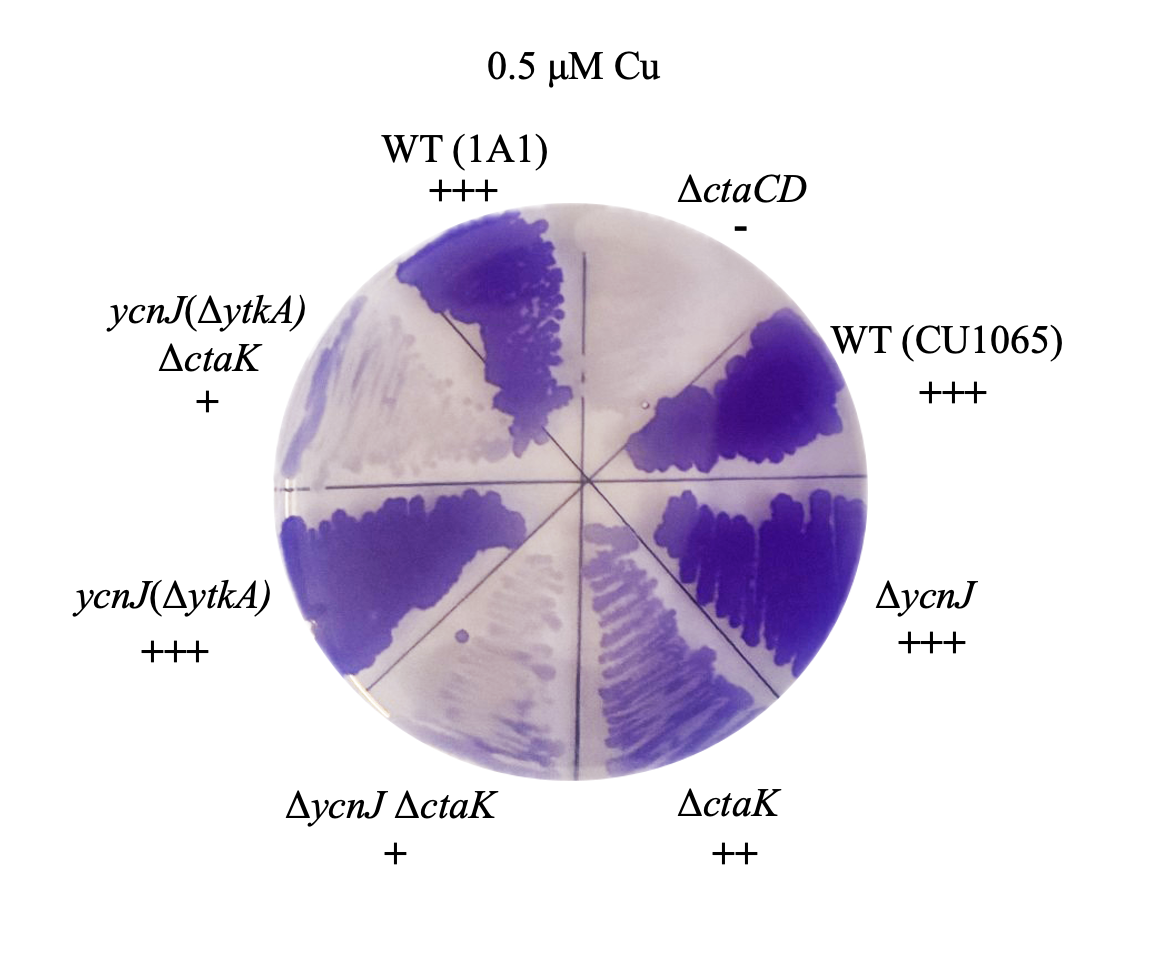


**Supplemental Figure 7. Quantification of TMPD staining intensities.** Growth of WT (1A1), Δ*ctaCD* (LUT3)*,* WT (CU1065), Δ*ycnJ*, Δ*ctaK*, Δ*ycnJ* Δ*ctaK*, *ycnJ*(Δ*ytkA*), and *ycnJ*(Δ*ytkA*) Δ*ctaK* on a TBAB agar plate with 0.5 μM CuSO_4_ added. Staining intensities displayed are linked to -, +, ++, and +++. This metric was used to assign values in Table 1 and Table S3.


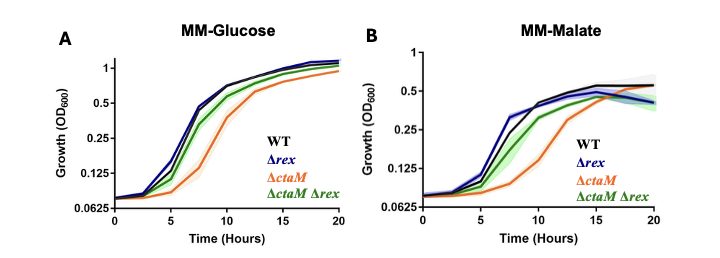


**Supplemental Figure 8. Δ*ctaM* has a growth defect in minimal media that can be overcome by derepressing Cyd*.*** Growth of WT, Δ*rex,* Δ*ctaM,* and Δ*ctaM* Δ*rex* in (A) MM-glucose (B) MM-malate. Results are from 3 biological replicates with the shaded region representing the standard deviation.


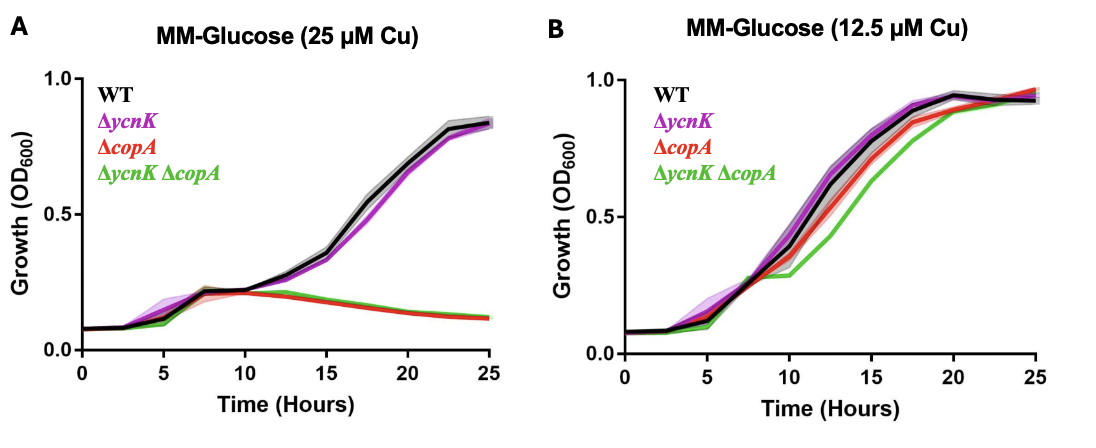


**Supplemental Figure 9. Δ*ycnK* Δ*copA* does not have increased sensitivity to Cu.** Growth of WT, Δ*ycnK,* Δ*copA,* and Δ*ycnK* Δ*copA* in MM-glucose supplemented with (A) 25 μM Cu and (B) 12.5 μM Cu. Results are from 3 biological replicates with the shaded region representing the standard deviation.


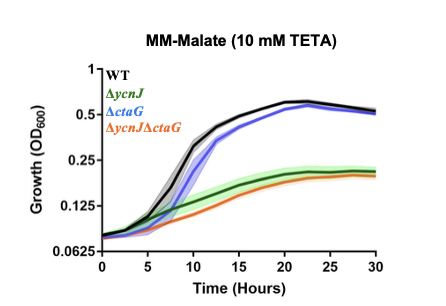


**Supplemental Figure 10. YcnJ can function independently of CtaG.** Growth of WT, Δ*ycnJ,* Δ*ctaG,* and Δ*ycnJ* Δ*ctaG* in MM-malate supplemented with 10 mM TETA. Results are from 3 biological replicates with the shaded region representing the standard deviation.

**Supplementary References:**

1. Sachla AJ, Luo Y, Helmann JD. 2021. Manganese impairs the QoxABCD terminal oxidase leading to respiration-associated toxicity. Mol Microbiol 116:729-742.

2. de Oliveira Silva YR, Barnes G, Zheng D, Zhitnitsky D, Geathers SJ, Peters SC, Szalai VA, Helmann JD, Fisher OS. 2025. Copper acquisition in *Bacillus subtilis* involves Cu(II) exchange between YcnI and YcnJ. J Biol Chem 301:110480.

3. Bengtsson J, von Wachenfeldt C, Winstedt L, Nygaard P, Hederstedt L. 2004. CtaG is required for formation of active cytochrome *c* oxidase in *Bacillus subtilis*. Microbiology 150:415-425.
